# Identification of Regulatory circRNAs Involved in the Pathogenesis of Acute Myocardial Infarction

**DOI:** 10.3389/fgene.2020.626492

**Published:** 2021-02-03

**Authors:** Cuimei Zhao, Jingjing Liu, Wen Ge, Zhi Li, Mengwei Lv, Yipeng Feng, Xuebo Liu, Ban Liu, Yangyang Zhang

**Affiliations:** ^1^Department of Cardiology, Tongji Hospital, Tongji University School of Medicine, Shanghai, China; ^2^Department of Cardiology, Wuxi People’s Hospital Affiliated to Nanjing Medical University, Wuxi, China; ^3^Department of Cardiothoracic Surgery, Shuguang Hospital Affiliated to Shanghai University of TCM, Shanghai, China; ^4^Department of Cardiovascular Surgery, Jiangsu Province Hospital, The First Affiliated Hospital of Nanjing Medical University, Nanjing, China; ^5^Shanghai East Hospital of Clinical Medical College, Nanjing Medical University, Shanghai, China; ^6^Department of Cardiovascular Surgery, Shanghai East Hospital, Tongji University School of Medicine, Shanghai, China; ^7^The First Clinical Medical College, Nanjing Medical University, Nanjing, China; ^8^Department of Cardiology, Shanghai Tenth People’s Hospital, Tongji University School of Medicine, Shanghai, China

**Keywords:** acute myocardial infarction, circRNA, miRNA, functional characterization, runt-related transcription factor-1

## Abstract

**Background:**

Acute myocardial infarction (AMI) has high morbidity and mortality worldwide. However, the pathogenesis of AMI is still unclear, and the impact of circular RNAs (circRNAs) on AMI has rarely been recognized and needs to be explored.

**Materials and Methods:**

The circRNA array was applied to investigate the expression level of circRNAs in the blood samples of coronary arteries of three AMI patients and three normal persons. Principal component analysis (PCA) and unsupervised clustering analysis were performed to reveal the distinguished expression patterns of circRNAs. The miRNA expression profiles of AMI patients were identified from a public dataset from the Gene Expression Omnibus (GEO) database (GSE31568). The miRNA binding sites on the circRNAs were predicted by miRanda. The miRNA enrichment analysis and annotation tool were used to explore the pathways that the dysregulated circRNAs may participate in.

**Results:**

In total, 142 differentially expressed circRNAs, including 89 upregulated and 53 downregulated in AMI samples, were identified by the differential expression analysis. AMI patients had quite different circRNA expression profiles to those of normal controls. Functional characterization revealed that circRNAs that had the potential to regulate miRNAs were mainly involved in seven pathways, such as the Runt-related transcription factor-1 (RUNX1) expression and activity-related pathway. Specifically, hsa_circRNA_001654, hsa_circRNA_091761, hsa_circRNA_405624, and hsa_circRNA_406698 were predicted to sponge four miRNAs including hsa-miR-491-3p, hsa-miR-646, hsa-miR-603, and hsa-miR-922, thereby regulating RUNX1 expression or activity.

**Conclusion:**

We identified dysregulated blood circRNAs in the coronary arteries of AMI patients and predicted that four upregulated circRNAs were involved in the regulation of RUNX1 expression or activity through sponging four miRNAs.

## Introduction

Acute myocardial infarction (AMI) is a common life-threatening disease that is manifested as myocardial necrosis caused by a prolonged period of ischemia and hypoxia in coronary arteries (Hirschl et al., 1996). Early diagnosis and treatment are the keys to improve the survival and prognosis of AMI patients (Vogel et al., 2019). Although the mortality of AMI has decreased thanks to medical developments in the modern era, there is still no radical cure for AMI because of a poor understanding of its pathogenesis and underlying mechanisms (Sulo et al., 2019). Therefore, further experimental and clinical investigations are urgently needed to provide new targets for the therapy or diagnosis of AMI.

Circular RNAs (circRNAs) belonging to non-coding RNAs with a closed continuous loop are heterogeneous transcripts derived from reverse splicing, and they regulate gene expression through multiple mechanisms (Memczak et al., 2013; Aufiero et al., 2019). Although the functions of circRNAs are still elusive, a certain number of circRNAs have been validated as microRNA (miRNA) sponges to exert regulatory function. Under specific circumstances, they bind to target miRNAs, preventing their interaction with messenger RNA (mRNA), thus regulating gene expression and signaling pathways (Hansen et al., 2013; Aufiero et al., 2019). CircRNAs play important roles in cardiovascular diseases. For instance, the heart-related circRNA (HRCR) could prevent cardiac hypertrophy and heart failure through sponging miR-233 to regulate the apoptosis repressor with CARD (ARC) expression (Wang et al., 2016). Circ-Foxo3, which is highly expressed in myocardial samples of older mice, exerts regulatory roles in cellular senescence (Du et al., 2017). The first study on circRNA and AMI showed that myocardial infarction-associated circRNA (MICRA) in peripheral blood can predict the left ventricular function in patients with acute AMI (Vausort et al., 2016). Although more studies on circRNA and cardiovascular diseases have been reported, there are still few studies on the relationship between circRNAs and AMI.

Non-coding RNAs have been found to play important roles in the pathophysiological process of AMI. However, the existing studies only provide the functional circRNAs in animal AMI models or the overall dysregulated circRNAs in the peripheral blood of AMI (Sun et al., 2020; Zhang et al., 2020). Little research has probed into the predominant regulatory circRNAs in circRNA-miRNA networks that are involved in vital regulatory pathways for the pathogenesis of AMI. Moreover, the differential expressions of blood circRNAs in the coronary arteries of AMI patients, and normal controls have not been reported. Thus, we aimed to investigate the abnormally expressed circRNAs in coronary arteries to explore the potential circRNAs-miRNAs networks in AMI patients and to identify the critical regulatory circRNAs. This study provides novel therapeutic or diagnostic targets for further research.

## Materials and Methods

### Ethics Statement

Written informed consent was obtained from all enrolled participants before applying the clinical records. All protocols adopted here were approved by the Ethics Committee at Shanghai East Hospital (ID: 2019057).

### CircRNA Profiling From circRNA Array Analysis

Arraystar human circRNA array was applied to detect and quantify circRNAs in the six samples of three AMI patients and three normal controls. The clinical data of the six participants were summarized in Table 1.

**TABLE 1:** The clinical data of the six personals.

**No.**	**Age (years)**	**Gender**	**Killip**	**Coronary**	**Complicated**	**Coronary**	**Smoking**	**D-to-B**	**Drug-diluted Stent**	**Culprit artery**
				**angiography**	**diseases**	**disease**		**(minutes)**	**(number)**	
1	55	Male	I	Positive	Hypertension	STEMI	Yes	85	1	LAD
2	52	Male	I	Positive	Hypertension	STEMI	Yes	80	1	LAD
3	47	Male	I	Positive	Hypertension	STEMI	Yes	90	1	LAD
4	49	Male	I	Negative	Hypertension	Normal	Yes	0	0	Negative
5	53	Male	I	Negative	Hypertension	Normal	Yes	0	0	Negative
6	55	Male	I	Negative	Hypertension	Normal	Yes	0	0	Negative

*STEMI, ST-segment elevation myocardial infarction; D-to-B, door-to-balloon time; LAD, left anterior descending coronary artery.*

Blood samples were collected from the coronary arteries of six participants [three with ST-elevation myocardial infarction (STEMI) and three without AMI] undergoing percutaneous coronary intervention (PCI) due to undiagnosed chest pain, and they were then prepared according to the arraystar’s standard protocols. Total RNA was extracted from whole blood with TRIzol reagent and tested for purity and concentration on Nanodrop 3000. After purification with an RNase-Free DNase set, RNAs were treated with RNase R to digest and remove linear RNAs. Next, microarray hybridization was conducted according to the manufacturer’s instructions, and the array images were obtained and analyzed in Agilent supporting software. CircRNAs were identified by performing an annotation strategy from the microarray dataset. The circRNA data were categorized by using linear or circular when a circRNA absenting or presenting in a sample. The categorized circRNAs were suitable for a generalization of principal component analysis, which generates a combined plot showing both patients and circRNAs closer together. An R programming tool was applied to standardize the array profile. The differential expression analysis was conducted by student *t*-test and fold change methods.

### The microRNA Expression Data From Gene Expression Omnibus (GEO) Database

The normalized miRNA expression data of blood samples from 18 AMI patients and 18 normal controls were downloaded from GEO database with accession GSE31568. The differentially expressed miRNAs were identified using the GEO2R analysis tool under a filter condition with adjusted *P* < 0.05 and fold change (FC) ≥2.

### Prediction of miRNA Binding Sites on the circRNAs

The miRNA binding sites on the circRNAs were predicted by miRanda (Miranda et al., 2006) with default options. Specifically, the miRNA and circRNA sequences were collected from the miRbase (Griffiths-Jones et al., 2008)^1^ and circRNA microarray annotation file, respectively.

### Functional Characterization of circRNAs by miRNA Enrichment Analysis

The miRNA enrichment analysis was conducted on the web server of miRNA Enrichment Analysis and Annotation Tool (miEAA)^2^ under a filter condition with adjusted *P* < 0.05. The interaction network between circRNAs and miRNAs was visualized by Cytoscape3.7.2.

### Principal Component Analysis (PCA) and Hierarchical Clustering

Principal component analysis was conducted based on the expression profiles of all circRNAs and implemented in R packages FactoMineR and Facto Extra. Hierarchical clustering was conducted on the differentially expressed circRNAs and visualized by the R package heatmap.

### Statistical Analysis

Statistical analyses were conducted by Student’s *t*-test, one-way analysis of variance (ANOVA), and a Tukey-Kramer multiple comparison test on R programming. All data are shown as mean ± standard deviation (SD).

## Results

### CircRNA Expression Profiles in AMI Patients and Controls

The blood samples were collected from the coronary arteries of three STEMI patients and three normal controls during PCI, and the clinical characteristics are summarized in Table 1. Little difference was found in age, gender, underlying diseases, or smoking history between the two groups.

**FIGURE 1:**
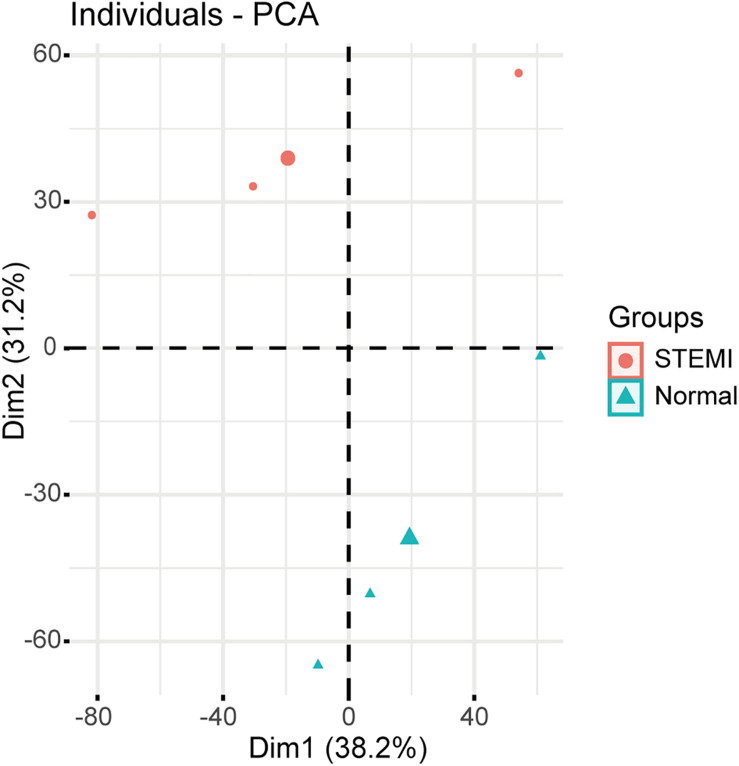
Relative distances between samples from AMI patients (red symbol) and controls (green symbol) by PCA. The top two principal components of the circRNA expression profiles.

Principal component analysis based on circRNA expression profiles was performed to investigate whether the expression patterns of circRNA between AMI patients and normal controls can be distinguished. A clear separation between the AMI group and the control group was observed (Figure 1), suggesting that AMI patients had quite different circRNA expression profiles from the normal controls and that circRNAs may potentially regulate the pathogenesis of AMI. These data strongly imply that microarrays along with PCA are probably effective approaches for distinguishing AMI patients and normal people.

### Identification of Differentially Expressed circRNAs

**FIGURE 2:**
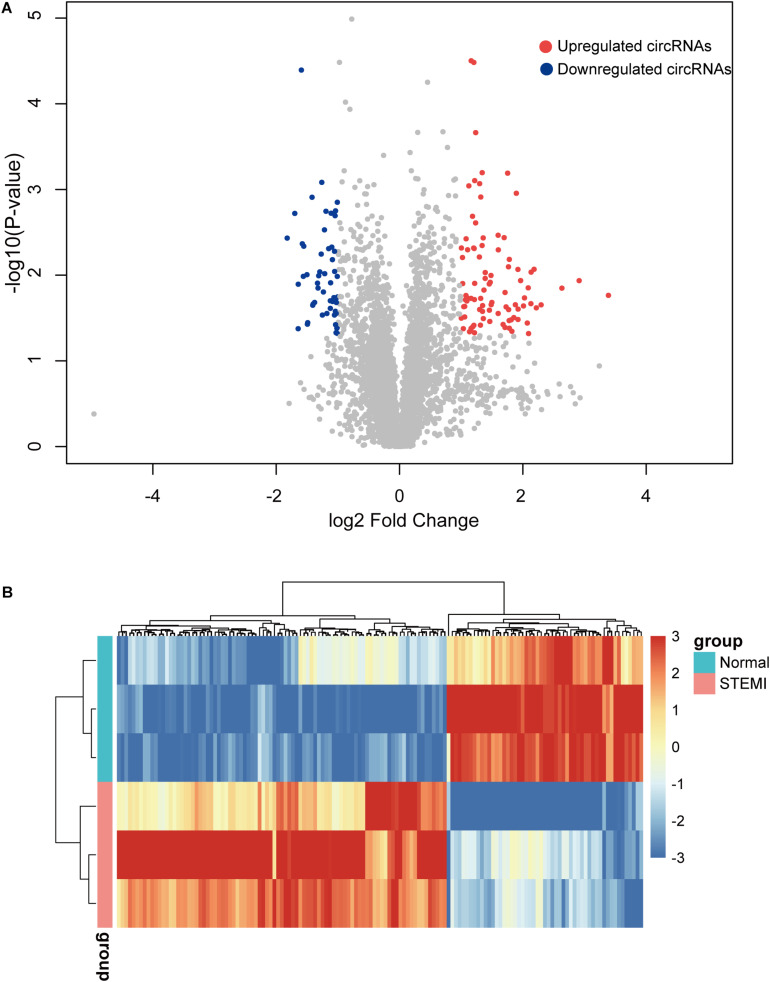
Differentially expressed circRNAs between AMI patients and controls. **(A)** The volcano plot of 142 circRNAs with a significant difference between groups. **(B)** Heatmap of dysregulated circRNAs by hierarchical clustering. *N* = 3, *P* < 0.05. ctrl, normal control (the same below).

To further identify the dysregulated circRNAs in AMI patients, we conducted differential expression analysis on the circRNA expression profiles. Specifically, a total of 142 differentially expressed circRNAs were identified by microarray with fold change >2 and adjusted *p* < 0.05, including 89 upregulated and 53 downregulated circRNAs compared with the control group (Figure 2A). Consistently, the hierarchical clustering revealed that AMI patients and normal controls can be classified by the differentially expressed circRNAs, and which were significantly different between AMI patients and normal controls (Figure 2B), suggesting these circRNAs were involved in the progress of AMI.

### CircRNA-miRNA Interaction Network Analysis

**FIGURE 3:**
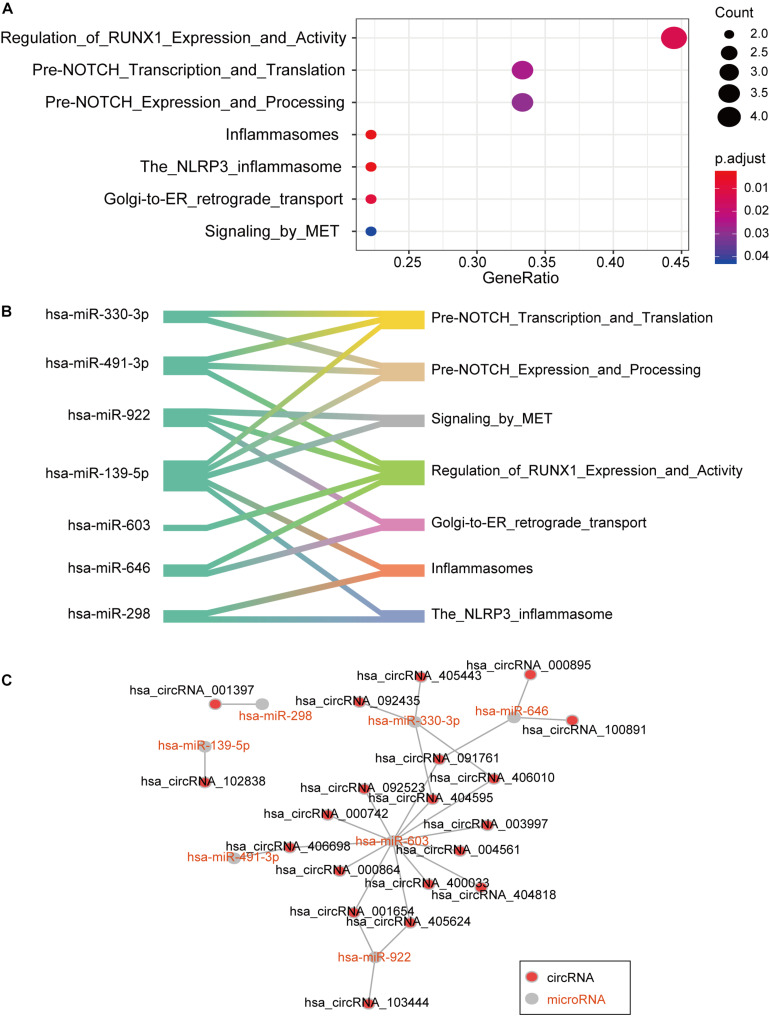
CircRNA-miRNA interaction networks of seven candidate miRNAs. **(A)** Signaling pathways enriched by the miRNAs interacting with circRNAs. **(B)** Networks between miRNAs and signaling pathways. **(C)** Interaction of seven candidate miRNAs with circRNAs.

The predominant function of circRNA is to regulate gene expression by sponging specific miRNAs (Hansen et al., 2013). To further explore the biological function of the dysregulated circRNAs, we identified the differentially expressed miRNAs between AMI patients and controls from the Gene Expression Omnibus (GEO) database with accession GSE31568. Specifically, 97 interactive miRNA-circRNA pairs were predicted by miRanda (Supplementary Table 1), including 97 circRNAs and 54 miRNAs. With the miRNAs potentially binding to circRNAs, we applied miRNA set enrichment analysis to characterize the function of circRNAs. Specifically, the circRNAs were primarily enriched in signaling pathways of mesenchymal-to-epithelial transition, Golgi-to-endoplasmic reticulum retrograde transport, the nucleotide-binding oligomerization domain-like receptor protein 3 (NLRP3) inflammasome, inflammasomes, pre-NOTCH expression and processing, pre-NOTCH transcription and translation, and regulation of Runt-related transcription factor-1 (RUNX1) expression and activity (Figure 3A). In addition, seven downregulated miRNAs in AMI patients were predicted to participate in regulating one or more signaling pathways (Figure 3B). Notably, hsa-miR-603, hsa-miR-330-3p, hsa-miR-646, and hsa-miR-922 can be sponged by multiple circRNAs (Figure 3C). These results indicated that the circRNAs may regulate RUNX1 expression and activity via sponging hsa-miR-603, hsa-miR-330-3p, hsa-miR-646, and hsa-miR-922.

### CircRNAs Involved in RUNX1 Regulation

**FIGURE 4:**
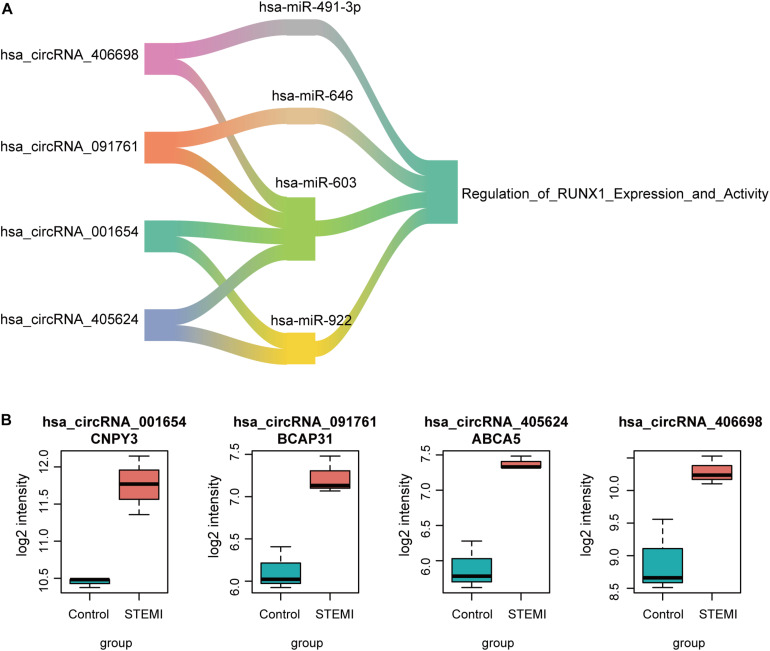
CircRNA-miRNA networks involved in RUNX1 regulation. **(A)** Interaction networks between targeted miRNAs in the regulation of RUNX1 and circRNAs. **(B)** Expressions of four candidate circRNAs in AMI and ctrl.

To further evaluate the regulatory circRNAs in circRNA-miRNA networks, we identified hsa_circRNA_001654, hsa_circRNA_091761, hsa_circRNA_405624, and hsa_circRNA_406698 as critical regulators in the networks, as they may sponge two or more miRNAs involved in the regulation of RUNX1 expression and activity (Figure 4A). Additionally, all of the four regulatory circRNAs were upregulated in AMI (Figure 4B). Particularly, three of the circRNAs were transcribed from the intragenic regions of protein-coding genes, including CNPY3, BCAP31, and ABCA5, while only hsa_circRNA_406698 was transcribed from intergenic regions. These results indicate that both intragenic and intergenic circRNAs have the potential to regulate RUNX1 expression and activity in the pathogenesis of AMI.

## Discussion

Circular RNAs were identified to be key regulators in the pathogenesis of AMI, and their abnormal expressions can significantly affect the disease progression (Zhang et al., 2020). In this study, we profiled the differentially expressed circRNAs in the blood of AMI patients and predicted the dysregulated circRNA-miRNA pairs involved in the regulation of AMI related signaling pathways. Further bioinformatic analysis identified four upregulated circRNAs in AMI patients, which might regulate RUNX1 expression or activity through sponging the miRNAs. Unlike previous studies, blood samples taken from the coronary artery were not conventional. However, we aimed to investigate the underlying regulatory function of circRNAs in AMI. Peripheral blood is relatively easy to collect and is more susceptible to the internal environment. Thus, the ncRNAs in readily available peripheral blood were thought to be more representative as biomarkers but less helpful for the understanding of molecular mechanism in the pathogenetic process of AMI. The analysis of the ncRNAs in coronary blood is more useful and direct for us to understand their regulatory function in AMI. Even so, we still think that both coronary blood and peripheral blood could be used to study the functional roles of circRNAs in AMI. In future analysis, the research of circRNAs in both coronary blood and peripheral blood should compensate for each other.

We presented four candidate circRNAs and four miRNAs probably involved in RUNX1 expression, which is critical in the regulation of various cellular processes, especially in hematopoiesis (Okuda et al., 2001; Ichikawa et al., 2013). The RUNX1 expression in adult hearts is significantly lower than in neonatal hearts (Eulalio et al., 2012). However, the re-activated RUNX1 in the infarct border zone of AMI patients has been widely recognized (McCarroll et al., 2018). RUNX1 upregulation is related to impaired cardiac contractile function and cardiac remodeling (McCarroll et al., 2018). *Runx1* knock-out can protect against adverse cardiac remodeling after AMI in mice (Riddell et al., 2020). Moreover, *Runx1* mRNA expression increases in the blood of AMI patients (Mao et al., 2017). The RUNX1 dysregulation after AMI occurs as early as 1 day post-AMI and can serve as a marker of early myocardial injury (Kubin et al., 2011). Our study demonstrates that hsa-miR-491-3p, hsa-miR-646, hsa-miR-603, and hsa-miR-922 are involved in regulation of RUNX1. As is well-known, miRNAs can inhibit translation to regulate gene expression via inducing the degradation of target mRNA (Bartel, 2004). Moreover, the predominant function of circRNAs is to bind to target miRNAs and prevent their interaction with mRNA, thus regulating gene expression. These four candidate miRNAs were down-expressed in AMI patients and were involved in regulatory networks with four upregulated circRNAs. Thus, hsa_circRNA_001654, hsa_circRNA_091761, hsa_circRNA_405624, and hsa_circRNA_406698 were identified to participate in regulating RUNX1 through targeting miRNAs. The interactions between circRNAs and microRNAs will be detected by luciferase reporter assay and RNA pull-down assay in our future research.

Acute myocardial infarction can be hardly distinguished from diseases presented chest pain, such as acute pulmonary embolism, acute pericarditis, and aortic dissection. Until now, invasive coronary angiography is still the gold standard in diagnosing AMI, and the biological markers in blood such as CK-MB and cTnT have some deficiencies in AMI diagnosis. Thus, more approaches and biological markers should be identified for the development of AMI diagnosis. Herein, the circRNA expression patterns in the blood of AMI patients were separated from normal controls, providing the probability of microarray along with PCA as effective approaches for distinguishing AMI patients and normal persons. CircRNAs are stably and highly conserved across species and are valuable biomarkers in diagnosing diseases (Zhang et al., 2018; Miao et al., 2019). Hsa_circRNA_001654, hsa_circRNA_091761, hsa_circRNA_405624, and hsa_circRNA_406698 were significantly increased in the blood of AMI and can serve as potential biomarkers for early diagnosis of AMI. Nevertheless, the potential role of these circRNAs as biomarkers should be further validated.

Apart from the regulation of RUNX1 expression and activity, other candidate pathways were also identified. Hsa-miR-330-3p, hsa-miR-491-3p, and hsa-miR-139-5p were all predicted to be involved in regulating the NOTCH pathway, a crucial mediator of cardiac repair and regeneration after AMI (Li et al., 2010; Wu et al., 2017). Moreover, the NOTCH signaling pathway is strongly connected with the RUNX1 expression in various diseases (Richard et al., 2013; Rodriguez-Caparros et al., 2019), which further indicates the profound roles of the candidate circRNAs in AMI regulatory networks. All the underlying signaling pathways are critical in cardiomyocytes of AMI and interact among cardiovascular diseases, which trigger us to speculate that the candidate circRNAs are released into the blood during myocardial damage and then exert feedback function on cardiomyocytes. In this research, we have predicted four circRNAs that could regulate the RUNX1 expression by sponging four miRNAs in AMI. Nevertheless, the underlying mechanisms of these circRNAs in the regulation of AMI warrant further investigation, which may facilitate the development of new targets for the treatment of AMI. To further investigate this biological process, we will test the expression of these ncRNAs in more coronary blood from AMI patients and explore their function in experimental models in future works.

## Limitations

There are some limitations in the study due to the limited research conditions: (1) The sample size in the circRNA array analysis was too small; (2) No experiment was conducted to verify the results of the circRNA array and bioinformatic analysis. In the future, more qualified samples should be enrolled into our analysis, and experiments will be carried out to validate our findings.

## Conclusion

We identified the differentially expressed circRNAs and potential circRNA-miRNA networks in AMI patients. Four upregulated circRNAs (hsa_circRNA_001654, hsa_circRNA_091761, hsa_circRNA_405624, and hsa_circRNA_406698) in the blood of AMI patients exerted regulatory function on RUNX1 expression or activity through sponging four downregulated candidate miRNAs (hsa-miR-491-3p, hsa-miR-646, hsa-miR-603, and hsa-miR-922).

## Data Availability Statement

The data presented in the study are deposited in the GEO database, and the accession number is GSE160717.

## Ethics Statement

The studies involving human participants were reviewed and approved by the Ethics Committee at Shanghai East Hospital. The patients/participants provided their written informed consent to participate in this study.

## Author Contributions

CZ conducted circRNA array analysis. JL performed GEO2R analysis. WG performed miRanda analysis. CZ and JL performed miRNA enrichment analysis. ZL and XL performed PCA analysis and hierarchical clustering analysis. ML and YF performed the data analysis. YZ, BL, and XL designed the experiments. CZ, JL, and WG wrote the manuscript. ML collected the blood sample. All authors read and approved the final manuscript.

## Conflict of Interest

The authors declare that the research was conducted in the absence of any commercial or financial relationships that could be construed as a potential conflict of interest.

## Footnotes

1 http://www.mirbase.org/

2 https://ccb-compute2.cs.uni-saarland.de/mieaa2/

## Supplementary Material

Supplementary Table 1 Identification of miRNA-circRNA pairs. Differentially expressed circRNAs identified in microarray analysis and dysregulated miRNAs identified in GSE 31568 between AMI and ctrl by application of miRanda; 97 circRNAs have binding sites with miRNAs.
