# Supplementary material for: Identification of Regulatory circRNAs Involved in the Pathogenesis of Acute Myocardial Infarction
Source: Front Genet. 2021 Feb 3;11:626492. doi: 10.3389/fgene.2020.626492 (PMC7886988; doi:10.3389/fgene.2020.626492)
Supplement: Supplementary file 2 [file Data_Sheet_1.docx]

Supplementary Material

## Table S1 footnote

**Table S1** Identification of miRNA-circRNA pairs. Differentially-expressed circRNAs identified in microarray analysis and dysregulated miRNAs identified in GSE 31568 between AMI and ctrl, by application of miRanda; 97 circRNAs have binding sites with miRNAs.
